# Herpetic Pruritus

**Published:** 1843-11

**Authors:** 


*Herpetic Pruritus.*—Dr. BAROSCH has cured a case of herpetic pruritus of the perineum and scrotum, which for twelve years resisted various remedies, and caused the greatest discomfort to the patient, by the following solution:—℞. Iodine, gr. xv., hydriod. potass. ℈ij.; solve in aq. distill. ℥v. alcohol, ℥j. M. This solution applied to the parts, in three weeks effected a complete cure.—*Amer. Journ. of the Med. Sci.*, from *Gaz. Méd. de Paris*, Jan. 28th, 1843, from *Oesterreichische Med. Wochens.*

